# A Dual-Branch Spatiotemporal Framework with Dynamic Weighted Permutation Entropy for Short-Window Motor Imagery EEG Decoding

**DOI:** 10.3390/s26134101

**Published:** 2026-06-27

**Authors:** Jiaju Wang, Haiqiang Yang

**Affiliations:** 1 School of Automation, Qingdao University, Qingdao 266071, China; wangjiaju@qdu.edu.cn; 2 Shandong Key Laboratory of Industrial Control Technology, Qingdao 266071, China; 3 Qingdao Key Laboratory of Embodied Intelligence and Robot Control, Qingdao 266071, China

**Keywords:** EEG, motor imagery, brain–computer interface, short-window decoding, dynamic weighted permutation entropy, spatiotemporal modeling

## Abstract

Decoding short-window electroencephalography (EEG) signals is critical for low-latency brain–computer interfaces (BCIs), yet current models struggle to extract robust features under high cross-subject variability and low signal-to-noise ratios. To address this, we propose a spatiotemporal decoding framework integrating dynamic weighted permutation entropy (DWPE) with a hybrid neural network. We introduce DWPE to quantify nonlinear dynamic complexity while retaining amplitude information. These features are subsequently processed by a cascaded convolutional neural network (CNN) and bidirectional long short-term memory (BiLSTM) architecture with spatial attention, enabling the simultaneous extraction of topological patterns and temporal dependencies. The framework was evaluated on three public motor imagery datasets (hBCI, BCI Competition IV-2a, and IV-2b) using a fixed 3 s window. Empirical results demonstrate that our approach achieves an average accuracy of 84.35% and an AUC of 0.8821 on the hBCI dataset, significantly outperforming current representative recent baselines (*p* < 0.01). Ablation studies confirm that integrating DWPE yields a 3.89% accuracy improvement over the spatial–temporal backbone alone. With a single-sample inference time of 20.94 ms and an estimated total decision latency of approximately 3.02 s under the 3 s window setting, the proposed method provides a favorable balance between decoding accuracy and computational efficiency for short-window and near-online BCI applications.

## 1. Introduction

Electroencephalography (EEG) is a noninvasive neurophysiological signal that captures neural population activity with high temporal resolution, and it has been widely adopted as a fundamental modality in brain–computer interface (BCI) systems. By decoding EEG signals, BCIs enable direct inference of user intentions without relying on peripheral neuromuscular pathways, thereby supporting a wide range of applications such as neurorehabilitation, assistive control, intelligent interaction, and human–machine collaboration [1,2]. Among various BCI paradigms, motor imagery (MI) is particularly attractive, as it elicits task-related cortical activity through imagined movements without requiring physical execution. This property makes MI suitable for practical scenarios including rehabilitation training, prosthetic control, and wheelchair navigation, and it has become a representative direction for short-time window BCI research [3].

Despite substantial progress in MI-based EEG decoding, achieving stable performance in real-world online systems remains challenging. EEG signals are inherently characterized by low signal-to-noise ratio, strong nonstationarity, and significant inter-subject variability, leading to considerable distribution shifts across subjects and trials [4]. In addition, MI-related neural activity is often contaminated by background EEG, electromyographic (EMG), electrooculographic (EOG), and environmental noise, making discriminative information difficult to extract reliably within short time intervals. Moreover, practical BCI systems require not only high decoding accuracy but also low decision latency and stable near-online responses. Traditional approaches typically rely on long temporal windows to accumulate sufficient information, which may improve offline performance but inevitably introduce delays that degrade interaction efficiency. Therefore, extracting discriminative features under short-time window constraints remains a critical challenge.

In recent years, deep learning methods have provided new perspectives for MI-based EEG decoding. Convolutional neural networks (CNNs) can automatically learn local temporal patterns from raw EEG signals, significantly reducing the need for handcrafted features [5,6]. Recurrent neural networks, particularly long short-term memory (LSTM) models, further capture temporal dependencies in sequential EEG data [7]. More recently, graph convolutional networks (GCNs) have been introduced to model spatial relationships by representing EEG channels as graph structures, thereby capturing topological dependencies among brain regions [8]. In addition, attention mechanisms and Transformer-based architectures have been applied to emphasize informative temporal segments and model long-range dependencies, leading to improved decoding performance [9,10,11]. Overall, these deep models have demonstrated strong capability in spatiotemporal representation learning [12]. Recent studies have further improved MI-EEG decoding through advanced deep learning frameworks, demonstrating enhanced robustness and generalization across subjects [13,14,15].

However, a key limitation of existing approaches lies in their predominant focus on temporal waveforms and spatial structures, while the nonlinear complexity embedded in EEG signals is often not explicitly modeled. In practice, MI-related neural activity involves dynamic reorganization of cortical processes, which is reflected not only in spatiotemporal interactions but also in signal complexity, irregularity, and local state transitions. Particularly in short-time window scenarios, waveform-based features are more susceptible to noise, whereas changes in signal complexity may provide earlier and more robust indicators of neural state transitions [16,17]. Consequently, relying solely on implicit feature learning through deep networks may be insufficient to capture these critical dynamics, thereby limiting decoding performance and robustness. Recent studies have further shown that entropy-based and connectivity-aware representations can complement deep learning models by providing additional information about EEG nonlinear dynamics, thereby improving robustness under noisy and short-window conditions [18,19,20]. Incorporating prior knowledge, such as spectral features or complexity-based representations, can provide complementary inductive biases and improve performance under low signal-to-noise conditions [21,22].

To better characterize nonlinear EEG dynamics, entropy-based measures have been widely investigated. Among them, permutation entropy (PE) and its variants have attracted considerable attention due to their computational efficiency and robustness to noise [23,24]. Compared with entropy measures such as Rényi entropy, PE is more suitable for short-time window EEG analysis because it relies on ordinal patterns rather than explicit probability density estimation, making it more robust under limited observations. Moreover, its ordinal-pattern representation provides a natural basis for the amplitude weighting and dynamic updating mechanisms adopted in the proposed DWPE framework [25,26,27]. By quantifying the uncertainty of local ordinal patterns, PE captures signal complexity beyond conventional amplitude-based representations. To further enhance its physiological relevance, weighted permutation entropy (WPE) incorporates amplitude information into ordinal patterns [28]. In addition, dynamic entropy analysis captures temporal variations in signal complexity, enabling better characterization of nonstationary EEG signals [29]. Despite these advances, existing entropy-based methods still face challenges in short-time window scenarios, including instability under limited data, lack of dynamic updating mechanisms, and limited integration with deep learning frameworks.

To overcome the above limitations, this paper proposes a dynamic weighted permutation entropy (DWPE)-enhanced dual-branch spatiotemporal framework for short-time window EEG decoding. The proposed method jointly models spatiotemporal representations and nonlinear complexity. Specifically, a deep spatiotemporal branch is designed to extract hierarchical features from raw EEG signals, capturing local temporal dynamics, spatial relationships, and long-range dependencies. In parallel, a complexity branch extends classical permutation entropy by incorporating amplitude weighting and dynamic recursive updating, resulting in a DWPE representation that characterizes both local fluctuations and temporal evolution of EEG complexity. The two types of features are then fused to form a unified representation, improving decoding accuracy, robustness, and efficiency under short-time window conditions.

The main contributions of this work are summarized as follows:

- A dynamic weighted permutation entropy (DWPE) method is proposed to enhance complexity modeling, integrating amplitude weighting and recursive updating to achieve a better balance between temporal resolution, stability, and physiological interpretability.
- A dual-branch spatiotemporal learning framework is developed, which explicitly fuses deep spatiotemporal features with dynamic complexity representations to jointly model temporal, spatial, and nonlinear characteristics of EEG signals.
- Extensive experiments are conducted on public datasets (hBCI, BCIC-IV-2a, and BCIC-IV-2b), demonstrating that the proposed method consistently outperforms representative recent baseline approaches in terms of accuracy, AUC, latency, and short-window adaptability.

The paper is organized as follows. Section 2 introduces the datasets, preprocessing procedures, and the proposed DWPE-enhanced framework. Section 3 presents the experimental setup and evaluation methodology. Section 4 reports the experimental results and ablation studies. Section 5 discusses the effectiveness and limitations of the proposed method. Finally, Section 6 concludes the paper and outlines future research directions.

## 2. Methodology

### 2.1. Dataset Description

Three publicly available motor imagery EEG datasets were used to evaluate the generalization capability of the proposed method, namely the hBCI dataset, BCI Competition IV-2a, and BCI Competition IV-2b. To ensure a consistent binary classification setting across datasets, only the left-hand and right-hand motor imagery trials were used in this study.

The open access EEG–NIRS hybrid BCI dataset (hBCI) released by Shin et al. [30] contains EEG recordings from 29 healthy subjects (14 males and 15 females, mean age 28.5±3.7 years). EEG signals were acquired from 30 active electrodes arranged according to the international 10–5 system at 1000 Hz. The dataset includes three motor imagery tasks: left hand, right hand, and both feet. Each subject completed three sessions, with 20 trials per class in each session. Each trial consisted of a visual cue (2 s), motor imagery (10 s), and rest (15–17 s). In this study, only left- and right-hand trials were retained, resulting in 3480 trials for binary classification.

The BCI Competition IV-2a dataset (BCIC-IV-2a) [31] contains EEG recordings from 9 subjects using 22 Ag/AgCl electrodes placed according to the 10–20 system at 250 Hz. It includes four motor imagery tasks: left hand, right hand, both feet, and tongue. Each subject completed two sessions on different days, with six runs per session and 48 trials per run (12 trials per class). Each trial consisted of fixation (0–2 s), a directional cue (1.25 s), and motor imagery (2–6 s). In this study, only left- and right-hand trials were retained, resulting in 2592 trials for binary classification.

The BCI Competition IV-2b dataset (BCIC-IV-2b) [32] includes EEG recordings from 9 right-handed subjects. Signals were collected using three bipolar channels (C3, Cz, and C4) under the 10–20 system, with a sampling rate of 250 Hz. A binary motor imagery task (left hand versus right hand) was adopted. Each subject participated in five sessions, including two sessions without feedback and three sessions with feedback. Each session consisted of ten runs, with 20 trials per run (10 trials per class). Each trial followed a sequence of fixation (0–3 s), cue presentation (1.25 s), and motor imagery (3–6.5 s). In total, 9000 trials were included.

### 2.2. EEG Data Preprocessing

A standardized preprocessing pipeline was applied to the raw EEG signals to improve signal quality and enhance motor imagery-related representations. The signals were first downsampled to 200 Hz to reduce computational complexity while preserving essential neural information. Subsequently, a band-pass filter of 8–30 Hz was applied to retain the μ (8–13 Hz) and β (13–30 Hz) rhythms closely associated with motor imagery and to suppress baseline drift and high-frequency noise. The complete preprocessing pipeline consisted of downsampling, band-pass filtering, ICA-based artifact removal, baseline correction, channel selection, trial segmentation, and sliding-window data augmentation.

To further improve signal reliability, artifact components, including electrooculographic (EOG) and electromyographic (EMG) activities, were removed using independent component analysis (ICA), a widely adopted method for EEG artifact correction [33]. Baseline correction was then performed to compensate for signal drift during recording. Channel selection was adapted to the electrode configuration of each dataset. For the hBCI dataset, eight sensorimotor electrodes, including FCC3h, FCC4h, FCC5h, FCC6h, CCP3h, CCP4h, CCP5h, and CCP6h, were selected as informative channels. For the BCIC-IV-2a dataset, eight sensorimotor channels surrounding the C3/C4 motor cortex region (FC3, FC1, FC2, FC4, CP3, CP1, CP2, and CP4) were selected using the same sensorimotor-region selection strategy. For the BCIC-IV-2b dataset, only the two lateral motor-related bipolar channels, C3 and C4, were retained for left- versus right-hand motor imagery classification, because they are more directly related to lateralized sensorimotor activity, whereas Cz mainly reflects midline activity and was therefore excluded to maintain a consistent lateral motor-region selection strategy.

To accommodate short-window decoding, EEG segments were extracted according to the motor imagery period defined in each dataset protocol. The original trials were first split into training and testing sets, and sliding-window segmentation was then performed separately within each set to avoid overlap-induced information leakage. Following previous preprocessing practice on the same motor imagery dataset, a 3 s sliding window with a 1 s step was used for data augmentation, which balances ERD/ERS-related information preservation and response latency while increasing the number of training samples. A consistent preprocessing framework was applied across all datasets, while dataset-specific channel configurations and trial timings were retained.

### 2.3. Dual-Branch Spatiotemporal Feature Learning Framework

**Figure 1:**
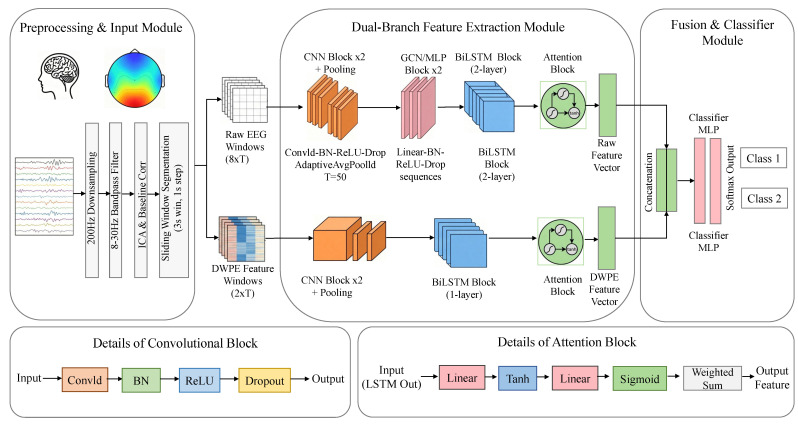
DWPE-enhanced dual-branch EEG classification framework. The two horizontal blocks at the bottom show the detailed structures of the convolutional block and the attention block used in the main architecture.

To integrate spatiotemporal dynamics and nonlinear complexity in EEG signals, we develop a dual-branch feature learning framework, as shown in Figure 1. The framework takes raw EEG signals and dynamic weighted permutation entropy (DWPE) features as complementary inputs and fuses them for final classification.

In the proposed architecture, the raw EEG branch first captures local temporal dynamics using a multi-scale residual one-dimensional convolutional module. Convolutional kernels with different receptive fields operate in parallel to extract temporal patterns at multiple scales, particularly those associated with μ and β rhythms. Residual connections are incorporated to facilitate gradient propagation and stabilize deep feature learning. The resulting representations are then processed by an adaptive graph convolutional module, where EEG channels are modeled as nodes in a graph. This module learns data-driven functional connectivity between channels, enabling effective modeling of the underlying spatial topology.

To further model temporal dependencies, a gated bidirectional long short-term memory (BiLSTM) network is employed to capture long-range sequential information. By leveraging contextual relationships within sliding windows, the BiLSTM enhances the representation of temporal dynamics. In addition, an attention mechanism is introduced to adaptively reweight features across different time steps, allowing the model to focus on the most informative segments relevant to motor imagery tasks.

In parallel, the DWPE branch provides complementary information from a nonlinear dynamics perspective. Specifically, it encodes the temporal evolution of signal complexity and introduces it as an auxiliary discriminative feature. The outputs from the two branches are then fused via feature concatenation, forming a unified representation that integrates deep spatiotemporal features and dynamic complexity characteristics. This representation is subsequently fed into a fully connected layer followed by a Softmax classifier for final task prediction.

By jointly learning from these complementary branches, the proposed framework captures temporal, spatial, and nonlinear complexity information in a unified manner, while maintaining a moderate computational cost. This design improves decoding robustness and stability, particularly under short-time window conditions.

### 2.4. DWPE-Based Complexity Modeling

#### 2.4.1. Classical Permutation Entropy

Classical permutation entropy (PE) quantifies signal complexity by measuring the uncertainty of local ordinal patterns in a time series, and is widely adopted due to its computational efficiency and robustness to noise. Given a one-dimensional EEG time series $x=\{x_{1},x_{2},\ldots ,x_{N}\}$, with embedding dimension *m* (set to 5 in this study) and time delay τ (set to 1), phase-space reconstruction is first performed. The occurrence probabilities of all m! permutation patterns are denoted as $p(\pi_{k})$, and the permutation entropy is defined as

(1) $$H_{PE}=-\sum_{k=1}^{m!}p\left(\pi_{k}\right)\log_{2}p\left(\pi_{k}\right)$$

The value of PE ranges from 0 to $\log_{2}\left(m!\right)$, where a larger entropy value indicates a higher degree of randomness in the signal. However, PE relies solely on ordinal pattern statistics and ignores amplitude-related information. Moreover, it is typically computed within a fixed window, which limits its ability to capture rapidly varying temporal characteristics in motor imagery EEG signals. To address these limitations, a dynamic weighted permutation entropy (DWPE) formulation is introduced.

#### 2.4.2. Dynamic Weighted Complexity Modeling

To address the limitations discussed above, two mechanisms, namely amplitude weighting and dynamic recursive updating, are introduced to construct the dynamic weighted permutation entropy (DWPE). The underlying idea is that, within a short-time window, amplitude variations of EEG signals (e.g., motor imagery-related ERD/ERS patterns) tend to emerge earlier than changes in ordinal structures, while signal complexity evolves continuously over time. Therefore, an effective complexity measure should simultaneously account for amplitude intensity and support online updating.

Specifically, to incorporate local fluctuation intensity into permutation probabilities, an amplitude weight is assigned to each embedded vector. This weight is computed as the mean absolute difference between consecutive sampling points, which reflects the local variation strength of the signal segment. Based on this formulation, the probability of each permutation pattern is further adjusted using the corresponding amplitude weights, as defined below:

(2) $$p_{w}\left(\pi_{k}\right)=\frac{\sum_{i\in S_{k}}\sum_{j=1}^{m-1}\left|x_{i+(j-1)\tau }-x_{i+j\tau }\right|}{\sum_{i=1}^{N-(m-1)\tau }\sum_{j=1}^{m-1}\left|x_{i+(j-1)\tau }-x_{i+j\tau }\right|},$$

where $S_{k}$ denotes the index set of embedded vectors corresponding to the permutation pattern $\pi_{k}$. The normalization condition $\sum_{k}p_{w}\left(\pi_{k}\right)=1$ is satisfied by construction. Through this weighting strategy, segments with stronger amplitude fluctuations contribute more to the entropy value, thereby linking the complexity measure to the intensity of neural activity in the cerebral cortex.

To further capture the continuously time-varying characteristics of EEG signals and support online processing, a combination of sliding window and exponential moving average is employed to update the weighted probability distribution. Let the instantaneous weighted probability distribution of the current window (length L=100 points, corresponding to 0.5 s) be denoted as $q_{t}$, and the dynamic probability from the previous time step as $P_{t-1}$. The update rule is defined as

(3) $$P_{t}=\alpha \cdot q_{t}+\left(1-\alpha \right)P_{t-1},\;\alpha \in \left(0,1\right],$$

where α=0.6 is the smoothing coefficient, which balances historical information and responsiveness to the current window. A larger α increases sensitivity to recent changes but also amplifies noise effects, whereas a smaller α improves stability at the cost of slower response. The initial condition is set as $P_{1}=q_{1}$. This recursive formulation only requires storing the probability vector from the previous step, leading to efficient computation.

Based on the dynamic probability distribution $P_{t}$, the dynamic weighted permutation entropy at time *t* is defined as

(4) $$H_{\mathrm{DWPE}}\left(t\right)=-\sum_{k=1}^{m!}P_{t}\left(\pi_{k}\right)\log_{2}P_{t}\left(\pi_{k}\right)$$

By traversing the entire EEG segment with a sliding window of fixed step size, a dynamic complexity sequence $\left\{H_{\mathrm{DWPE}}\left(1\right),H_{\mathrm{DWPE}}\left(2\right),\ldots \right\}$ is obtained. This sequence characterizes the continuous evolution of EEG complexity from the resting state to the motor imagery state, covering transition, task maintenance, and recovery phases.

The key parameter settings of the proposed DWPE are summarized as follows: embedding dimension m=5, time delay τ=1, window length L=100 samples, corresponding to 0.5 s after downsampling to 200 Hz, smoothing coefficient α=0.6, and sliding step Δt=1. The 0.5 s DWPE window was selected to capture local dynamic complexity changes within short EEG segments while avoiding excessive fluctuation caused by very short windows. The smoothing coefficient α=0.6 was used to balance historical probability information and current-window information, thereby improving stability while preserving responsiveness to rapid motor imagery-related EEG changes. These parameter choices were further validated through the parameter sensitivity analysis in Section 4.5.

### 2.5. Spatiotemporal Feature Learning Module

The spatiotemporal feature learning architecture serves as the backbone of the proposed dual-branch decoding framework, enabling hierarchical extraction and integration of spatiotemporal representations from raw EEG signals. The architecture consists of four sequential modules, including a multi-scale residual one-dimensional convolutional module, an adaptive graph convolutional module, a gated bidirectional long short-term memory (BiLSTM) module, and an attention-based temporal modeling module.

These components are organized into a progressive pipeline that performs local temporal feature extraction, spatial topology modeling, long-range temporal dependency learning, and adaptive emphasis on informative patterns. Through this hierarchical process, discriminative features associated with motor imagery are gradually refined. By jointly exploiting temporal dynamics and spatial correlations in EEG signals, the proposed module provides high-quality spatiotemporal representations, which are subsequently fused with complexity features for final decoding.

#### 2.5.1. Multi-Scale Residual One-Dimensional Convolutional Module

The multi-scale residual one-dimensional convolutional module acts as the front-end component of the spatiotemporal feature learning framework, enabling robust extraction of local temporal patterns from raw EEG signals at different resolutions. Motor imagery-related neural activity is primarily reflected in the μ (8–13 Hz) and β (13–30 Hz) frequency bands. Meanwhile, EEG signals are characterized by nonstationarity, low signal-to-noise ratio, and rapid transient variations. Under these conditions, a single-scale convolution is insufficient to capture fast fluctuations, rhythmic patterns, and long-term trends simultaneously.

To address this limitation, a parallel multi-branch convolutional structure is adopted, where kernels with different receptive field sizes are used to extract features at multiple temporal scales. This design enables joint modeling of transient variations, core rhythmic components, and longer-range temporal dependencies.

Let the input multichannel EEG signal be $X\in R^{C\times T}$. To reduce the influence of amplitude variation and baseline drift, the input is first standardized as

(5) $$\hat{X}=\frac{X-\mu }{\sigma },$$

where μ and σ denote the mean and standard deviation along the temporal dimension, respectively.

After multi-scale feature extraction, the outputs from different branches are concatenated along the channel dimension. A 1×1 convolution is then applied to fuse features and perform channel-wise transformation. To mitigate gradient vanishing and feature degradation in deep networks while preserving useful signal characteristics, a residual connection is introduced. Specifically, the standardized input is directly added to the fused features, which enhances representation learning and stabilizes network training.

(6) $$F_{\mathrm{res}}={\mathrm{Conv}}_{1\times 1}\left(\left[F_{1},F_{2},F_{3}\right]\right)+\hat{X}$$

Batch normalization and ReLU activation are applied after each convolutional layer to stabilize feature distributions, accelerate convergence, and enhance nonlinear representation capability. Following residual fusion, dropout regularization is further introduced to reduce the risk of overfitting. The module ultimately outputs local temporal features that capture multi-scale temporal information and exhibit improved robustness to noise, providing effective inputs for subsequent spatial modeling.

#### 2.5.2. Adaptive Graph Convolutional Module

The adaptive graph convolutional module is designed to model the spatial structure of electrode channels and to learn functional relationships among them, serving as a core component for spatial feature extraction in EEG decoding. In contrast to traditional approaches that construct graph structures based on predefined anatomical layouts, this module adopts a data-driven strategy to learn the adjacency matrix adaptively. This design reduces the impact of spurious spatial correlations caused by electrode placement and allows the model to better capture meaningful interactions between brain regions.

In this module, each electrode channel is treated as a graph node, and the temporal features extracted from the previous stage are used as node attributes. A learnable parameter matrix is employed to compute pairwise similarities between nodes, based on which the adjacency matrix is constructed and sparsified. To ensure numerical stability and facilitate gradient propagation during graph convolution, self-loops are added to the adjacency matrix, followed by symmetric normalization:

(7) $$\hat{A}={\hat{D}}^{-\frac{1}{2}}\left(A+I\right){\hat{D}}^{-\frac{1}{2}},$$

where *A* denotes the learned adjacency matrix, *I* is the identity matrix, and $\hat{D}$ represents the corresponding degree matrix.

After normalization, graph convolution is applied to aggregate and propagate neighborhood information. A residual connection is further introduced to preserve the temporal features learned in the previous stage, mitigating the loss of low-level information during spatial modeling.

(8) $$F_{\mathrm{spatial}}=\mathrm{ReLU}\left(\hat{A}F_{\mathrm{res}}W+F_{\mathrm{res}}\right)$$

This design enables the model to capture spatial activation patterns of the sensorimotor cortex during motor imagery, as well as functional interactions across brain regions, thereby improving the physiological relevance and discriminative capability of the learned spatial features.

#### 2.5.3. Gated Bidirectional LSTM and Attention Temporal Modeling Module

The gated bidirectional long short-term memory (BiLSTM) and temporal attention module is positioned at the final stage of the spatiotemporal feature learning architecture. It is designed to model long-range temporal dependencies and to adaptively emphasize informative temporal segments, thereby addressing the limitations of convolutional networks in long-sequence modeling. Motor imagery is an inherently dynamic neural process that evolves over time, and the corresponding EEG patterns exhibit pronounced temporal dependencies. Therefore, relying solely on convolutional operations with local receptive fields is insufficient to capture the global temporal dynamics of the signal.

This module employs a bidirectional LSTM to encode temporal sequences in both forward and backward directions, thereby leveraging contextual information within the sliding window to better capture the full temporal evolution of motor imagery, including initiation, maintenance, and termination phases. Based on the temporally encoded features, a temporal attention mechanism is further introduced to assign higher weights to task-relevant time steps while suppressing noise and redundant information. The attention weights are computed as follows:

(9) $$\alpha_{t}=\frac{\exp\left(v_{a}^{⊤}\tanh\left(W_{a}H_{t}+b_{a}\right)\right)}{\sum_{k=1}^{T}\exp\left(v_{a}^{⊤}\tanh\left(W_{a}H_{k}+b_{a}\right)\right)}$$

The temporal features are aggregated using attention weights to obtain the final spatiotemporal representations. These representations integrate multi-scale temporal information, spatial correlations, and long-range temporal dependencies, resulting in improved discriminative capability and robustness. They can be effectively fused with the DWPE complexity features, providing reliable support for the final motor imagery classification.

## 3. Experimental Setup

### 3.1. Experimental Protocol

**Comparison Experiments.** To provide a comprehensive comparison, nine baseline models are selected, including four representative deep learning architectures and five widely used or recently proposed methods for motor imagery EEG decoding. All baseline models are implemented under the same preprocessing pipeline, dataset partitioning strategy, and evaluation metrics to ensure a fair comparison. The baseline models used for comparison are summarized in Table 1.

**Ablation Studies.** Two groups of ablation experiments are conducted on the hBCI dataset. In the first group, spatial graph convolution, bidirectional LSTM, and attention mechanisms are progressively incorporated into the backbone network to construct the baseline model (without DWPE), followed by integration of DWPE to form the complete model. The evaluated variants include *Conv Only*, *CNN & Spatial*, *CNN & BiLSTM*, *CNN & Spatial & BiLSTM*, *Baseline*, and *Proposed*. In the second group, DWPE features are used independently with classical classifiers, including support vector machines (SVMs), multilayer perceptrons (MLPs), and random forests (RFs), to assess their standalone discriminative capability.

**Sensitivity Analysis.** Sensitivity analysis is conducted on two key parameters of the dynamic weighted permutation entropy (DWPE), namely the smoothing coefficient α and the embedding dimension *m*. The values of α are selected from {0.1,0.3,0.5,0.6,0.7,0.9}, while the embedding dimension *m* is varied within {3,4,5,6,7}.

All other hyperparameters are kept fixed. Five-fold cross-validation is adopted to evaluate the influence of these parameters, using average classification accuracy and area under the curve (AUC) as performance metrics.

### 3.2. Training Strategy

In this study, stratified sampling is adopted to split the dataset of each subject into training and testing sets with a ratio of 85% to 15%, ensuring consistent class distributions. This split was performed at the original trial level before sliding-window segmentation, so that overlapping windows from the same trial were not shared between the training and testing sets.

A fixed random seed is used, and non-deterministic CUDA operations are disabled to improve reproducibility. For each subject and each model, the experiment was conducted once under the fixed random seed, and the reported results in Table 2 were averaged across subjects.

The model is optimized using the AdamW optimizer with an initial learning rate of $1\times 10^{-3}$, which is gradually reduced to $1\times 10^{-5}$ using a cosine annealing schedule. The loss function is a class-balanced cross-entropy. During training, gradient clipping (maximum norm of 1.0) and early stopping (triggered if the validation accuracy does not improve for 15 consecutive epochs) are applied to stabilize training. The batch size is set to 64, and the maximum number of training epochs is 200.

The model is implemented in PyTorch and trained on an NVIDIA GPU. Classification accuracy and area under the curve (AUC) are monitored on the validation set, and the best-performing model is retained. During testing, inference time is measured by feeding samples individually.

### 3.3. Evaluation Metrics

The performance of the proposed model is evaluated using the following four metrics:

**Classification Accuracy (ACC):** The ratio of correctly classified samples to the total number of samples, reflecting the overall classification performance.

**Area Under the Curve (AUC):** The area under the receiver operating characteristic (ROC) curve, which evaluates the model’s ability to distinguish between classes. For binary classification tasks, AUC is computed for each class and averaged, while for multi-class tasks, a one-versus-rest strategy is adopted.

**Inference Time per Sample:** The time required for a single forward pass of the model on one test sample (in milliseconds). This metric is measured with a batch size of 1, repeated 100 times, and averaged to assess real-time decoding performance.

**Information Transfer Rate (ITR):** A metric that quantifies the communication efficiency of the brain–computer interface system, defined as follows:

(10) $$\mathrm{ITR}=\frac{60}{T}\left(\log_{2}N+P\log_{2}P+\left(1-P\right)\log_{2}\frac{1-P}{N-1}\right),$$

where *N* is the number of classes, *P* is the classification accuracy, and *T* is the decision time. In this study, the motor imagery task is binary classification; therefore, N=2. The decision time *T* is defined as the sum of the EEG acquisition window length and the average single-sample inference time. Cue presentation, feedback duration, and inter-trial rest periods are not included in *T*.

### 3.4. Hyperparameter Settings

The key hyperparameters of the proposed model are summarized as follows:

**Data preprocessing:** Downsampling to 200 Hz, band-pass filtering within 8–30 Hz, independent component analysis (ICA) for artifact removal, selection of eight sensorimotor electrode channels, and a sliding window of 3 s with a step size of 1 s.

**DWPE features:** Embedding dimension m=5, time delay τ=1, window length L=100 (corresponding to 0.5 s), smoothing coefficient α=0.6, and sliding step size of 1 sample.

**Multi-scale convolutional module:** Kernel sizes of the three parallel branches are 3, 5, and 7, respectively. Each branch contains 16 output channels with a stride of 1, and padding is applied to preserve the temporal dimension.

**Adaptive graph convolutional module:** The number of graph convolution layers is set to 1, the hidden dimension is 64, and the sparsification threshold of the adjacency matrix is 0.5.

**Bidirectional LSTM module:** The hidden dimension is 128, the number of layers is 1, and the dropout rate is 0.5.

**Attention mechanism:** The dimensions of queries, keys, and values are all set to 128.

**Classifier:** A fully connected layer maps the fused features to the number of classes, followed by a Softmax activation function.

**Training hyperparameters:** Initial learning rate of $1\times 10^{-3}$, reduced to $1\times 10^{-5}$ using cosine annealing, batch size of 64, maximum of 200 epochs, early stopping with a patience of 15 epochs, gradient clipping with a norm of 1.0, and weight decay of $1\times 10^{-4}$.

All baseline models adopt the hyperparameter settings recommended in their original studies and are retrained and evaluated under the same data splits for fair comparison.

## 4. Results

### 4.1. Comparison Experiments

To comprehensively evaluate the decoding performance of the proposed method under different data distributions, task complexities, and acquisition conditions, experiments are conducted on three public motor imagery datasets, namely hBCI, BCIC-IV-2a, and BCIC-IV-2b. The preprocessing pipeline is kept consistent across all datasets, while channel selection is adapted according to the electrode configuration of each dataset.

Table 2 presents the average classification accuracy (ACC), area under the receiver operating characteristic curve (AUC), and F1 score of the proposed method and the baseline models across the three datasets. As all tasks are binary classification, AUC is directly computed based on the predicted probabilities of the positive class. To further evaluate statistical significance, paired *t*-tests are conducted at the subject level by comparing the accuracy of the proposed method with each baseline model, with the significance level set to α=0.05. In Table 2, the best results are highlighted in bold, and statistical significance is indicated by markers (* for p<0.05, ** for p<0.01).

As shown in Table 2, the proposed method achieves consistently competitive performance across all three datasets. To better reflect inter-subject variability, the results in Table 2 are reported as mean ± standard deviation across subjects. On the hBCI dataset, it attains the highest accuracy of 84.35% with an AUC of 0.8821, which significantly outperforms the best baseline, ADF-GCN (79.33%, p<0.01). On the BCIC-IV-2a dataset, the proposed method reaches an accuracy of 79.20% with an AUC of 0.8159, surpassing ADF-GCN (77.99%, p<0.05). Similarly, on the BCIC-IV-2b dataset, it achieves an accuracy of 76.20% with an AUC of 0.7980, again outperforming ADF-GCN (74.95%, p<0.05). Overall, the proposed method consistently exceeds all baseline models across multiple evaluation metrics, demonstrating strong generalization ability under varying task complexities and electrode configurations.

**Figure 2:**
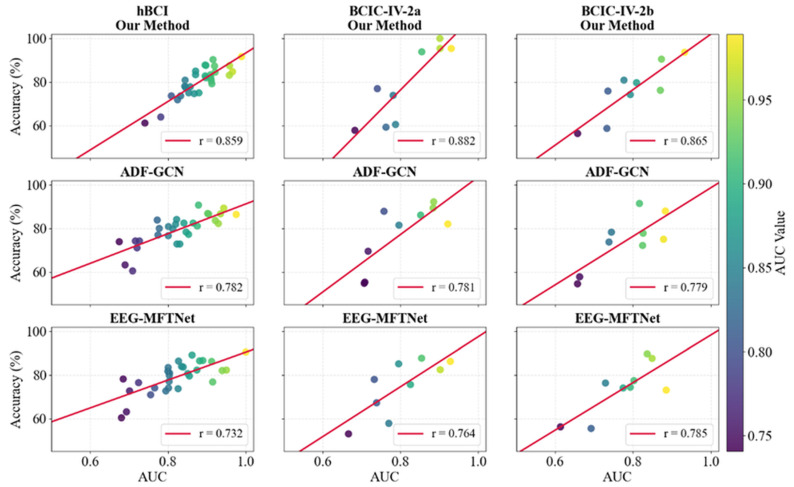
Individual subject-level correlation between test accuracy and AUC across three datasets.

To further analyze subject-level consistency, Figure 2 illustrates the distribution of test accuracy and AUC for individual subjects across the three datasets. The first row corresponds to the proposed method, while the second and third rows represent ADF-GCN and EEG-MFTNet, respectively. The three columns correspond to the hBCI, BCIC-IV-2a, and BCIC-IV-2b datasets. Each point denotes a subject, where the horizontal axis represents AUC and the vertical axis represents classification accuracy (%). The color of each point encodes the corresponding AUC value, and the red line indicates the fitted linear regression. The Pearson correlation coefficient *r* is also reported.

As shown in Figure 2, the proposed method exhibits a clear positive correlation between test accuracy and AUC across all three datasets. On the hBCI dataset, most subjects achieve accuracies above 75% and AUC values above 0.85, with a correlation coefficient of r=0.859 (p<0.001). Similarly, strong correlations are observed on BCIC-IV-2a (r=0.882, p<0.001) and BCIC-IV-2b (r=0.865, p<0.001).

These results indicate that the proposed method maintains stable discriminative performance across subjects, with AUC showing strong linear consistency with classification accuracy. In contrast, the baseline method ADF-GCN yields lower correlation coefficients of 0.782, 0.781, and 0.779 on the three datasets, respectively, while EEG-MFTNet achieves 0.732, 0.764, and 0.785, all of which are notably lower than those of the proposed method. This suggests that the proposed approach achieves stronger subject-level consistency and more reliable performance across individuals.

A small number of subjects (e.g., those with accuracy below 65% on hBCI) exhibit relatively lower performance. This phenomenon may be attributed to inter-subject variability in neural signals, differences in task engagement, or variations in recording conditions. Overall, the results in Figure 2 are consistent with the average performance reported in Table 2, further demonstrating that the proposed method achieves robust decoding performance for the majority of subjects and exhibits improved adaptability across different individuals.

**Figure 3:**
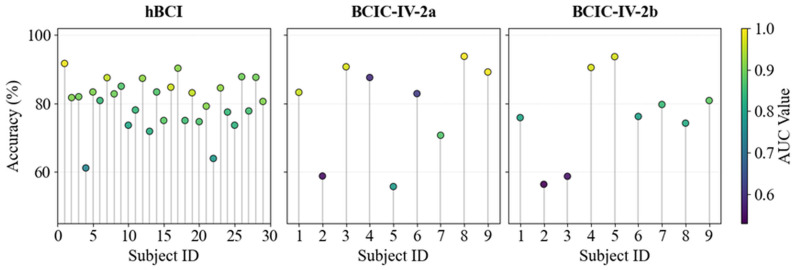
Individual test accuracy distribution of the proposed method across three datasets.

Figure 3 illustrates the distribution of subject-level test accuracy of the proposed method across the three datasets. Each subplot is presented as a dot plot, where the vertical position of each point represents the subject’s test accuracy, and the color encodes the corresponding AUC value. Vertical reference lines are included to facilitate visual alignment.

The results reveal noticeable variability in performance across subjects. On the hBCI dataset (29 subjects), accuracy ranges from 61% to 92%, with an average of 84.35%. Subjects 16, 17, and 26 achieve accuracies above 90%, whereas subject 4 attains only 61.11%, reflecting substantial inter-subject differences in neural signal characteristics. On the BCIC-IV-2a dataset (9 subjects), accuracy varies from 55% to 94%, with an average of 79.20%. Subjects 3, 8, and 9 exceed 90%, while subject 5 achieves 55.67%. On the BCIC-IV-2b dataset (9 subjects), accuracy ranges from 56% to 94%, with an average of 76.20%, where subjects 4 and 5 achieve accuracies above 90%, and the highest reaches 93.65%.

Furthermore, the color distribution (AUC values) shows a consistent positive relationship with accuracy, reinforcing the reliability of the evaluation. Overall, Figure 3 indicates that the proposed method achieves strong decoding performance for most subjects and maintains good adaptability across individuals, although inter-subject variability remains an important factor influencing performance.

To further examine whether the low-accuracy subjects are specific to the proposed method or reflect a common challenge across models, we compared their performance with representative baseline methods in Figure 2. The results show that subjects with relatively low accuracy under the proposed method also tend to exhibit lower performance under ADF-GCN and EEG-MFTNet. This suggests that the reduced performance of these subjects is more likely related to subject-specific EEG characteristics, such as weak or delayed ERD/ERS responses, lower signal-to-noise ratio, inconsistent motor imagery engagement, or dataset-specific recording differences, rather than being caused by the proposed framework alone. Nevertheless, the proposed method still improves the overall subject-level consistency, as indicated by the higher accuracy–AUC correlation compared with the baseline methods.

Overall, the proposed method achieves stable and competitive performance across three public datasets with varying scales and task complexities, demonstrating strong generalization ability and adaptability across subjects.

### 4.2. Ablation Experiments

To validate the effectiveness of each component in the proposed model, systematic ablation experiments are conducted in this section from two perspectives: the backbone network structure and the DWPE complexity branch. First, three classical classifiers, including support vector machines (SVMs), multilayer perceptrons (MLPs), and random forests (RFs), are applied using only DWPE features to examine whether these features contain independent discriminative information. Second, the EEG backbone network is progressively constructed to analyze the impact of the convolutional feature extraction module, spatial topology modeling module, temporal dependency modeling module, and attention mechanism on performance. Finally, the DWPE feature branch is introduced on top of the complete backbone network to evaluate the performance gain it provides. All experiments are conducted on the hBCI dataset, and the results are reported in Table 3.

Table 3 shows that when only DWPE features are used with shallow classifiers, the performance remains relatively low, with the best average accuracy of 63.18% obtained by random forests (RF) and an AUC of 0.6441, only slightly above random guessing. These results suggest that DWPE features alone do not provide sufficient class-discriminative information and are not adequate for independently performing motor imagery decoding.

During the progressive construction of the backbone network, the model achieves limited performance when only the convolutional module is included, indicating that local temporal convolution alone is insufficient for extracting high-level discriminative features. After introducing the spatial topology modeling module, the performance improves, with accuracy increasing from 61.64% to 66.11% and AUC increasing from 0.6286 to 0.6358, suggesting that modeling spatial correlations among channels enhances EEG feature representation. With the addition of the BiLSTM module, both accuracy and AUC further increase, reaching 67.91% and 0.7367, respectively, highlighting the importance of long-range temporal dependency modeling in capturing the dynamic evolution of motor imagery. When the attention mechanism is incorporated, the performance improves substantially, with accuracy increasing to 80.46% and AUC increasing to 0.8587, indicating that different temporal segments contribute unequally to classification and that emphasizing informative time steps improves discriminative capability. Overall, the backbone network modules exhibit a clear hierarchical improvement trend, validating the effectiveness of the proposed spatiotemporal feature extraction pipeline.

After integrating DWPE features into the complete backbone network, the proposed model further increases the accuracy from 80.46% to 84.35% and improves the AUC from 0.8587 to 0.8821, while the inference time increases by only approximately 4.4 ms, from 16.52 ms to 20.94 ms, remaining within an acceptable range. This result demonstrates that the nonlinear complexity information captured by DWPE is complementary to the deep spatiotemporal features, and their integration leads to enhanced decoding performance. It also confirms the effectiveness of the proposed dual-branch framework, which introduces complementary information rather than merely increasing feature dimensionality.

### 4.3. Inference Efficiency and Latency Analysis

For short-window and near-online BCI applications, the model should maintain high classification accuracy while keeping computational latency low. This section evaluates the inference efficiency of the proposed method by jointly analyzing inference time per sample and classification accuracy. The experiments are conducted on a general-purpose computing platform equipped with an NVIDIA GPU with CUDA acceleration, with a batch size of 1 under PyTorch inference mode. The inference time per sample of all baseline models is measured under the same experimental conditions and analyzed together with classification accuracy across the three datasets.

**Figure 4:**
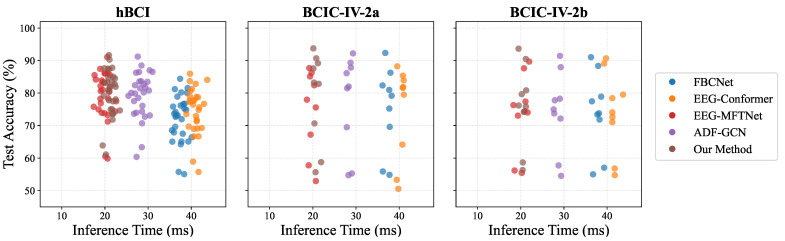
Trade-off between classification accuracy and single-sample inference time for different models across the three datasets.

Figure 4 presents the relationship between classification accuracy and inference time for different models across the three datasets, where the horizontal axis represents inference time and the vertical axis represents classification accuracy. Since lower inference time and higher accuracy are preferred, models closer to the upper-left region indicate a more favorable trade-off between efficiency and performance. The proposed method achieves the highest accuracy across the three datasets while maintaining a moderate inference time. On the hBCI dataset, it achieves an accuracy of 84.35% with an inference time of 20.94 ms. Among the baseline models, EEG-MFTNet and ADF-GCN achieve relatively close accuracies of 79.15% and 79.33%, respectively, while the proposed method exceeds them by 5.20 and 5.02 percentage points, respectively. At the same time, its inference time is lower than that of ADF-GCN and comparable to that of EEG-MFTNet. FBCNet and EEG-Conformer exhibit longer inference times with accuracies below 75%. Although MS-TSEFNet achieves the shortest inference time, its accuracy remains notably lower than that of the proposed method. These results indicate that the proposed approach achieves a more favorable balance between decoding accuracy and computational efficiency across different datasets, avoiding both the limited performance of lightweight models and the high latency of complex architectures.

Regarding computational cost, the proposed framework does not introduce a substantially higher burden than existing approaches. Since all baseline models were evaluated under the same preprocessing pipeline, data splits, batch size, and inference environment, the single-sample inference time comparison in Figure 4 provides a fair assessment of computational efficiency. In addition, the ablation results in Table 3 show that adding the DWPE branch increases the inference time from 16.52 ms to 20.94 ms, corresponding to an additional cost of only 4.42 ms, while improving the accuracy from 80.46% to 84.35% and the AUC from 0.8587 to 0.8821. This indicates that the performance improvement is achieved with limited additional overhead rather than by substantially increasing model complexity.

The overall system latency consists of the data acquisition window (3 s) and the model inference time (20.94 ms), resulting in a total delay of approximately 3.02 s. This latency is suitable for applications that do not require strict real-time responsiveness, such as rehabilitation assessment and non-critical control scenarios. For online closed-loop BCI systems requiring sub-second responses (e.g., drone control or high-speed typing), the time window can be further reduced, or causal convolutional structures can be adopted to replace the bidirectional LSTM.

Overall, the proposed method achieves a strong balance between decoding accuracy and inference efficiency, demonstrating its practical potential for real-world BCI deployment.

### 4.4. Effect of Time Window Length

In practical BCI applications, system response latency is largely determined by the data acquisition window length. To investigate the trade-off between decoding performance and decision latency under different window settings, experiments are conducted on the hBCI dataset (binary classification) using window lengths of 1 s, 2 s, and 3 s. All windows are aligned to the stimulus onset (0 s). The training and testing protocols remain consistent with those described in Section 3.1, with only the input window length being varied.

**Figure 5:**
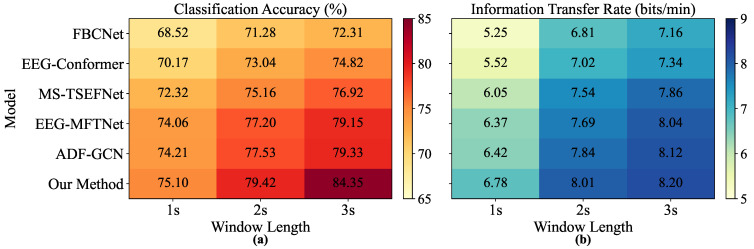
Heatmaps of classification accuracy (**a**) and information transfer rate (**b**) across window lengths.

Five representative methods, including FBCNet, EEG-Conformer, MS-TSEFNet, EEG-MFTNet, and ADF-GCN, are selected as baselines. Together with the proposed method, a total of six models are compared. Figure 5 illustrates the classification accuracy (a) and information transfer rate (b) under different window lengths in the form of heatmaps, where darker colors indicate better performance and numerical values are annotated in each cell. The ITR is computed according to the formulation in Section 3.3. Since the hBCI experiment is a binary motor imagery classification task, the number of classes is set to N=2. The decision time *T* is defined as the sum of the EEG window length and the average single-sample inference time. Other task-related intervals, such as cue presentation, feedback duration, and inter-trial rest periods, are not included in *T*. Therefore, the reported ITR reflects the decoding-stage communication efficiency under different window-length settings.

From Figure 5a, the accuracy of all models increases as the window length extends from 1 s to 3 s, reflected by the progressively darker colors across columns. The proposed method consistently achieves the highest accuracy under all three window settings, reaching 84.35% at the 3 s window and significantly outperforming the baseline models. When the window length is reduced to 2 s, the proposed method still maintains an accuracy of 79.42%, while all other models remain below 78%. Even under the extremely short 1 s window, it achieves 75.10%, exceeding ADF-GCN (74.21%) and the remaining baselines, demonstrating strong robustness under short-window conditions.

Figure 5b presents the variation of ITR across different window lengths. The proposed method shows a consistent increase in ITR, rising from 6.78 bits/min at 1 s to 8.01 bits/min at 2 s, and further to 8.20 bits/min at 3 s, indicating that the gain in accuracy effectively compensates for the increased decision time. In contrast, several baseline methods exhibit slower growth or even slight degradation in ITR from 2 s to 3 s, suggesting that their accuracy improvements are insufficient to offset the additional time cost. Overall, the proposed method achieves a favorable ITR of 8.01 bits/min at a 2 s window while reaching its highest accuracy at 3 s, enabling flexible selection of window length depending on application requirements, such as speed-oriented or accuracy-oriented scenarios.

### 4.5. Parameter Sensitivity

The proposed dynamic weighted permutation entropy (DWPE) serves as a core feature module for short-window motor imagery EEG decoding, where the smoothing coefficient α and the embedding dimension *m* are two key parameters that directly influence feature extraction quality and overall decoding performance. The smoothing coefficient α controls the balance between historical information and current window information in the dynamic recursive process, thereby affecting noise robustness and sensitivity to temporal variations. The embedding dimension *m* determines the granularity of ordinal pattern representation during phase-space reconstruction of EEG signals.

To systematically investigate the joint effect of these parameters, grid search experiments are conducted on the three datasets. Specifically, α is selected from {0.1,0.3,0.5,0.6,0.7,0.9}, and *m* is varied within {3,4,5,6,7}, resulting in 30 parameter combinations. For each configuration, all other network structures and training settings remain unchanged. Five-fold cross-validation is employed, and the average classification accuracy (ACC) is used as the evaluation metric.

**Figure 6:**
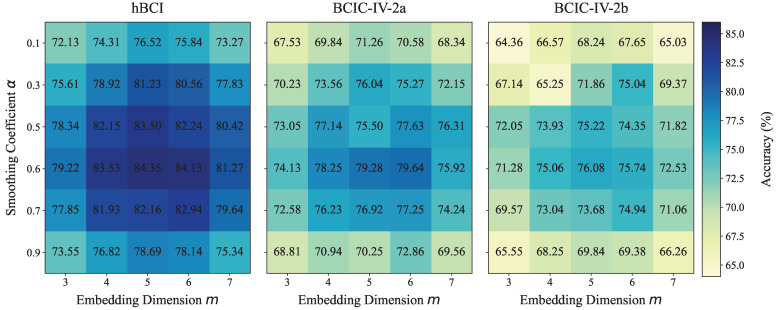
Parameter sensitivity heatmaps of DWPE on three datasets.

Figure 6 presents the heatmaps of classification accuracy for different (α,m) combinations across the three datasets, where darker colors indicate higher accuracy and the corresponding values are annotated in each cell. A clear unimodal distribution is observed on all datasets, with the optimal parameter region consistently centered around α=0.6 and m=5. Under this setting, the hBCI dataset achieves the highest accuracy of 84.35%, while BCIC-IV-2a and BCIC-IV-2b reach 79.20% and 76.20%, respectively, which are consistent with the average results reported in Table 2. This observation suggests that the optimal DWPE parameters remain stable across datasets with different distributions and task complexities.

When α deviates from 0.6, the accuracy on all datasets shows a decreasing trend. For small values of α (e.g., 0.1), the dynamic probability distribution relies excessively on historical information, which improves smoothing but slows down the response to rapid neural state transitions. Conversely, for large values of α (e.g., 0.9), the model places excessive emphasis on the instantaneous information from the current window, making the DWPE features more sensitive to noise and short-window fluctuations. Regarding the embedding dimension *m*, the optimal performance is consistently achieved at m=5. When *m* is too small (e.g., 3), the reconstructed phase space fails to sufficiently represent temporal ordinal patterns, while overly large values (e.g., 7) introduce redundant information and noise, increasing computational cost and the risk of overfitting. Notably, the performance degradation around the optimal region is relatively gradual. For instance, when α=0.5 or α=0.7 with m=5, the accuracy on hBCI remains at 83.5% and 82.16%, respectively, while BCIC-IV-2a and BCIC-IV-2b also maintain high performance. This indicates that DWPE exhibits good robustness within a relatively wide parameter range, which is beneficial for practical deployment across different scenarios.

Based on the results across the three datasets, α=0.6 and m=5 are selected as the default parameter configuration for DWPE. This setting achieves a favorable balance among noise suppression, dynamic responsiveness, feature granularity, and model robustness. It is validated as effective on the datasets considered in this study and provides a practical reference for parameter selection in other motor imagery EEG applications.

## 5. Discussion

This paper proposes a dual-branch spatiotemporal collaborative modeling method that integrates dynamic weighted permutation entropy (DWPE) for short-window motor imagery EEG decoding. Experimental results on three public datasets (hBCI, BCIC-IV-2a, and BCIC-IV-2b) show that the proposed method outperforms representative baseline models, including conventional deep learning architectures and recent EEG decoding methods, in terms of classification accuracy, AUC, and short-window adaptability. On the hBCI dataset with 29 subjects, the method achieves an average accuracy of 84.35%, an AUC of 0.8821, and an inference time within 21 ms. On BCIC-IV-2a and BCIC-IV-2b, the accuracies reach 79.20% and 76.20%, respectively. These results demonstrate strong generalization capability across different task complexities and electrode configurations, while maintaining a favorable balance between accuracy and efficiency.

The main contribution of this work lies in incorporating DWPE as an explicit complexity feature branch and fusing it with deep spatiotemporal representations. Ablation results show that introducing DWPE on top of the complete backbone network improves accuracy from 80.46% to 84.35% and AUC from 0.8587 to 0.8821. In contrast, when DWPE features are used alone with classical classifiers, including support vector machines (SVMs), multilayer perceptrons (MLPs), and random forests (RFs), the best average accuracy is only 63.18%, indicating limited discriminative capability when used independently. These results suggest that DWPE and deep features play complementary roles: the deep branch captures task-related temporal and spatial patterns, while DWPE provides amplitude-aware and dynamically updated complexity information. Compared with conventional PE and WPE, DWPE is more suitable for short-window MI-EEG decoding because it incorporates amplitude-related variations and dynamically updates the weighted ordinal-pattern distribution, enabling it to track rapid local complexity changes while reducing short-window fluctuations. This complementary mechanism is critical for maintaining robustness under low SNR and short decision windows.

From the perspective of backbone architecture, the contributions of individual modules exhibit a clear hierarchical progression. The multi-scale convolution captures temporal patterns associated with μ and β rhythms using different receptive fields. The adaptive graph convolution models functional connectivity among electrodes in a data-driven manner, reducing the limitations of fixed topological structures. The bidirectional LSTM captures the temporal evolution of motor imagery processes, and the attention mechanism highlights informative time steps. These components follow a progressive pipeline of local temporal enhancement, spatial relationship modeling, long-range dependency capture, and key information focusing, which is consistent with the intrinsic spatiotemporal characteristics of EEG signals. However, it should be noted that the bidirectional LSTM introduces non-causal dependencies, which limits its direct application in strictly real-time online systems.

In terms of near-online applicability and short-window adaptability, the proposed method adopts a fixed sliding window of 3 s, resulting in an initial system latency of approximately 3.02 s. While this latency is not suitable for sub-second closed-loop control, it may be suitable for applications such as rehabilitation assessment and non-critical interaction. Experiments with different window lengths show that when the window is reduced to 2 s, the accuracy remains at 79.42%, still exceeding all baseline methods. Even under a 1 s window, the accuracy reaches 75.1%. The rapid convergence of classification accuracy indicates that the model can achieve stable performance with limited signal accumulation, which is closely related to the sensitivity of DWPE to early-stage complexity variations. A fixed window strategy is adopted to ensure consistency and reproducibility across subjects and datasets, while avoiding additional hyperparameter tuning and overfitting risks associated with adaptive window schemes. Future work may explore lightweight individualized window calibration strategies to further improve adaptability without increasing computational cost.

Despite the promising performance, several limitations remain. First, the model still shows performance variability across subjects. For example, in the BCIC-IV-2a dataset, subject 5 achieves only 55.67% accuracy, and several subjects in the hBCI dataset also exhibit relatively low performance. According to the subject-level comparison in Figure 2, these low-performing subjects also tend to show reduced accuracy under existing baseline approaches, indicating that this phenomenon is mainly associated with inter-subject variability rather than being specific to the proposed framework alone. Possible causes include weak or delayed ERD/ERS responses, unclear sensorimotor rhythm modulation, lower signal-to-noise ratio, inconsistent motor imagery engagement, and dataset-specific recording differences. To alleviate this issue, future work will investigate subject-specific calibration, adaptive channel selection, individualized DWPE parameter tuning, and transfer learning or domain adaptation strategies to improve decoding performance for subjects with weak or unstable motor imagery patterns.

Future work will focus on addressing these limitations. Possible directions include developing subject-specific calibration strategies for DWPE parameters, incorporating transfer learning or domain adaptation to improve generalization, and replacing bidirectional LSTM with causal temporal models to enable real-time deployment. In addition, DWPE can be reformulated as a differentiable module for end-to-end learning, reducing reliance on manual parameter tuning. Further validation on clinical datasets and integration with multimodal signals, such as fNIRS and EMG, will also be explored to enhance practical applicability. Overall, this study provides a feasible framework for short-window motor imagery EEG decoding by combining complexity modeling with deep spatiotemporal feature learning, offering potential for short-window and near-online BCI applications.

## 6. Conclusions

In this study, we introduced a DWPE-guided spatiotemporal network tailored for short-window EEG decoding in motor imagery BCIs. By fusing amplitude-sensitive nonlinear complexity metrics, namely dynamic weighted permutation entropy (DWPE), with a CNN-BiLSTM backbone, the model systematically mitigates the temporal resolution limitations of traditional spatial filters. Extensive evaluations across the hBCI, BCIC-IV-2a, and BCIC-IV-2b datasets validate that the proposed approach outperforms recent comparative models, such as ADF-GCN and EEG-MFTNet, when processing 3 s EEG segments. Furthermore, ablation experiments confirm that the DWPE module explicitly improves feature discriminability, contributing to a 3.89% gain in classification accuracy. Beyond predictive performance, the proposed framework maintains computational efficiency, requiring only 20.94 ms per trial for inference. While the current 3 s fixed-window design ensures cross-subject reproducibility, future work will investigate subject-specific adaptive windowing strategies and evaluate the closed-loop deployment of this framework in asynchronous online BCI environments.

## Figures and Tables

**Table 1:** Summary of baseline models used for comparison.

Model	Main Characteristic
CNN [34]	Convolution-based EEG feature extraction
Transformer [35]	Self-attention-based temporal dependency modeling
Deep ConvNet [36]	End-to-end deep convolutional EEG decoding
TCNet [37]	Temporal convolutional sequence modeling
FBCNet [38]	Filter-bank convolutional feature learning
EEG-Conformer [39]	Convolution–Transformer hybrid EEG decoding
MS-TSEFNet [40]	Multi-scale spatiotemporal feature extraction
EEG-MFTNet [41]	Multi-scale feature fusion network
ADF-GCN [42]	Adaptive graph convolutional spatial modeling

**Table 2:** Overall performance of the proposed method on three public datasets.

Model	hBCI			BCIC-IV-2a			BCIC-IV-2b		
	ACC (%)	AUC	F1 (%)	ACC (%)	AUC	F1 (%)	ACC (%)	AUC	F1 (%)
CNN	68.47±12.83	0.7183±0.1172	68.31±12.76	69.64±11.65	0.7152±0.1084	69.52±11.58	68.71±10.92	0.7126±0.1015	68.59±10.85
Transformer	71.56±10.74	0.7469±0.0968	71.42±10.68	72.81±9.86	0.7437±0.0893	72.69±9.79	71.84±9.25	0.7391±0.0842	71.70±9.18
Deep ConvNet	70.13±11.52	0.7324±0.1041	70.02±11.45	71.38±10.47	0.7289±0.0952	71.24±10.41	70.69±9.83	0.7241±0.0890	70.53±9.76
TCNet	72.84±9.67	0.7592±0.0872	72.71±9.61	73.95±8.92	0.7546±0.0804	73.80±8.86	72.53±8.37	0.7482±0.0758	72.39±8.31
FBCNet	72.31±9.94	0.7582±0.0895	72.27±9.87	71.89±9.35	0.7542±0.0841	71.83±9.28	72.97±8.14	0.7601±0.0736	72.89±8.07
EEG-Conformer	74.82±8.36	0.7843±0.0754	74.75±8.29	74.50±7.81	0.7710±0.0698	74.42±7.74	73.77±7.26	0.7685±0.0651	73.59±7.19
MS-TSEFNet	76.92±7.25	0.7926±0.0653	76.81±7.18	75.30±6.74	0.7803±0.0605	75.18±6.67	73.06±7.61	0.7720±0.0684	73.02±7.54
EEG-MFTNet	79.15±6.43	0.8231±0.0579	79.02±6.36	78.34±5.92	0.8015±0.0528	78.26±5.85	74.36±7.05	0.7752±0.0632	74.27±6.98
ADF-GCN	79.33±6.27	0.8256±0.0564	79.21±6.20	77.99±6.15	0.8032±0.0546	77.83±6.08	74.95±6.68	0.7814±0.0601	74.83±6.61
**Our Method**	**84.35 ± 5.11** **	**0.8821 ± 0.0462** **	**84.32 ± 5.07** *	**79.20 ± 5.63** *	**0.8159 ± 0.0504** *	**79.15 ± 5.58** *	**76.20 ± 6.12** *	**0.7980 ± 0.0547** *	**76.17 ± 6.08** *

**Note:** Values are reported as mean ± standard deviation across subjects. Compared with the best-performing baseline methods, statistical significance is evaluated using paired *t*-tests, where * denotes p<0.05 and ** denotes p<0.01.

**Table 3:** Ablation study results on the hBCI dataset.

Model	ACC (%)	AUC	Inference Time (ms)
DWPE & SVM	60.56	0.6067	<1
DWPE & MLP	59.73	0.6038	<1
DWPE & RF	63.18	0.6441	<1
Conv-Only	61.64	0.6286	1.42
CNN & Spatial	66.11	0.6358	6.95
CNN & BiLSTM	67.91	0.7367	13.26
CNN & Spatial & BiLSTM	75.62	0.7945	15.47
Baseline (without DWPE)	80.46	0.8587	16.52
**Proposed (with DWPE)**	**84.35**	**0.8821**	**20.94**

## Data Availability

The datasets used in this study are publicly available. The hBCI dataset, BCI Competition IV-2a dataset, and BCI Competition IV-2b dataset can be obtained from their official repositories.
